# Comparison of long‐term use of prolonged‐release ropinirole and immediate‐release dopamine agonists in an observational study in patients with Parkinson's disease

**DOI:** 10.1002/pds.4986

**Published:** 2020-03-09

**Authors:** Usha Gungabissoon, Oksana Kirichek, Céline El Baou, Nicholas Galwey

**Affiliations:** ^1^ Department of Epidemiology GlaxoSmithKline (GSK) R&D Uxbridge UK; ^2^ Real World Data Analytics GSK R&D Uxbridge UK; ^3^ Medicines Research Centre Stevenage GSK R&D Hertfordshire UK

**Keywords:** adherence and persistence, dopamine agonist, dyskinesia, immediate release, impulse control disorders, levodopa, pharmacoepidemiology, prolonged‐release ropinirole

## Abstract

**Purpose:**

To estimate the risk of dyskinesia and impulse control disorders (ICDs) in patients with Parkinson's disease (PD) prescribed ropinirole prolonged‐release (R‐PR) compared to those prescribed immediate‐release dopamine agonists (IR‐DA) as monotherapy.

**Methods:**

PD patients initiating R‐PR or IR‐DA as monotherapy between 2008 and 2013 were identified on the Clinical Practice Research Datalink. The cohorts were propensity score matched on a 1:1 basis. The incidence of dyskinesia and ICD in each treatment cohort and the incidence rate ratios were calculated. Adherence to medication and time to levodopa initiation were also evaluated.

**Results:**

We identified 341 patients in each treatment cohort after propensity score matching. The baseline characteristics were generally comparable. Dyskinesia incidence in R‐PR and IR‐DA cohorts was 2.98 (95% CI: 0.74‐11.9) and 3.93 (95% CI: 0.98‐15.7) per 1000 person‐years, respectively (incidence rate ratio of R‐PR vs ID‐DA: 0.76, 95% CI: 0.11‐5.38). Less than five cases of ICD were identified and all occurred in the IR‐DA cohort. The patients in the R‐PR cohort remained on treatment for a significantly longer duration than those in the IR‐DA cohort (682 days vs 444 days; *P* < .0001) and had greater adherence to the medication. The median time to levodopa initiation was 417 days (IQR: 205‐736) in R‐PR vs 297 days (IQR: 111‐552) in IR‐DA cohort.

**Conclusions:**

The number of dyskinesia and ICD events was lower than expected, resulting in an underpowered study. A significantly longer persistence and greater adherence to medication was observed in patients receiving R‐PR compared to IR‐DA.

1

**KEY POINTS**

- The incidence of dyskinesia and impulse control disorders (ICDs) in Parkinson's disease patients who were prescribed prolonged‐release ropinirole (R‐PR) was compared with those prescribed an immediate‐release dopamine agonist (IR‐DA) in this observational study.
- The limited sample size and lower than expected number of dyskinesia and ICD events resulted in low power, making it inappropriate to draw conclusions from comparisons between the treatment cohorts for these outcomes.
- Patients prescribed R‐PR had significantly greater medication persistence and adherence compared to patients prescribed IR‐DA.
- A non‐significant delay in time to levodopa initiation in those treated with R‐PR vs IR‐DA cohort was observed.

## 2 INTRODUCTION

Parkinson's disease (PD) is an age‐related progressive neurodegenerative condition affecting approximately 1.5% of the global population above the age of 65 years.^1^ The recommended first‐line treatment option for patients with PD includes dopamine agonists (DA), levodopa and monoamine oxidase B (MAO‐B) inhibitors.^2^, ^3^ Both, dyskinesias and impulse control disorders (ICDs) are known consequences associated with the use of dopaminergic therapies in patients with PD.^4^, ^5^ Although levodopa is often considered the gold standard for treating PD, it is known to induce motor complications and dyskinesia.^6^, ^7^ A 5‐year study in early stage PD found that dyskinesia occurred in 45% of patients initially treated with levodopa compared to 20% of patients initially treated with ropinirole.^8^

Ropinirole (Requip™) is a potent non‐ergoline DA used either as monotherapy or in addition to levodopa for the management of early and advanced PD.^9^ The immediate‐release (IR) and prolonged‐release (PR) oral formulations were approved in the United Kingdom in 1996 and 2008, respectively. At the time of approval, limited data were available describing the outcomes associated with the long‐term use of ropinirole prolonged‐release (R‐PR). This observational study aimed to compare the incidence of dyskinesias and ICDs in patients with PD initiating R‐PR to those initiating an oral immediate‐release dopamine agonist (IR‐DA) as monotherapy using data from a UK primary care database. Additionally, adherence to and persistence with the medication were evaluated between the treatment cohorts. Further, time to levodopa initiation was measured since a delay in levodopa initiation may further delay the development of dyskinesia in patients with PD.^10^

## 3 METHODS

### 3.1 Study design

This study utilised a propensity score matched cohort design using data recorded in the Clinical Practice Research Datalink (CPRD). The population of interest was patients with PD who initiated R‐PR or an oral IR‐DA as monotherapy between January 2008 and December 2013.

### 3.2 Data source

The CPRD is an observational electronic health record database that contains data recorded as part of routine clinical care within UK primary care.^11^ The medical events and prescriptions generated from general practitioners (GPs) are coded using the Read and British National Formulary (BNF) coding systems, respectively.

### 3.3 Study population

Individuals aged ≥40 years were required to have ≥1 Read code for primary PD at the time of diagnosis. The PD diagnosis date was defined as the earliest date of a prescription for a dopaminergic therapy or Read code for PD or a PD symptom. Only patients registered at practices that were up to research standard were included, the CPRD recommends that this criteria is used to select research‐quality GP practices.^11^ In addition, eligible patients were required to have ≥2 prescriptions of the medication of interest and ≥12 months of registration prior to the index date (defined as the date of initiation of treatment plus 30 days).

Patients with a history of dyskinesia, ICD or levodopa use or evidence of secondary or drug‐induced Parkinsonism on or prior to the index date were excluded. Patients in the IR‐DA cohort with evidence of use of any prolonged‐release DA (ropinirole‐PR or pramipexole‐PR) prior to the index date were also excluded. Patients receiving oral ergot‐ or non‐ergot‐derived immediate release dopamine agonist formulations (ropinirole, bromocriptine, cabergoline, lisuride, pergolide and pramipexole) were included in the IR‐DA cohort.

Follow‐up commenced from the index date until the following (whichever was earliest): development of dyskinesia or ICD, discontinuation of therapy of interest plus 30 days or end of follow‐up on the CPRD, up to a maximum of 5 years of treatment.

The study protocol was approved by the UK's Independent Scientific Advisory Committee for Medicines and Healthcare Products Regulatory Agency database research. Confidentiality of the patients was maintained as per CPRD policy.^11^ The sponsor did not have access to potentially patient identifiable information.

Version 2017 Q3 of the CPRD was used. Statistical analysis was performed using SAS Version 9.2.

### 3.4 Propensity score matching

The R‐PR and IR‐DA cohorts were matched on a 1:1 basis using greedy matching.^12^, ^13^, ^14^ The following variables were included in the calculation of the propensity score: age at index date, gender, history of hypercholesterolemia, history of hypertension, history of diabetes, use of MAO‐B inhibitor prior to initiation, PD duration at initiation, number of GP visits in the 12 months prior to treatment initiation and the Charlson comorbidity score.^15^ Standardised differences were calculated between the baseline covariates for both cohorts before and after matching to assess balance; a standardised difference of ≤0.20 was considered as a threshold for adequate balance between the cohorts.^16^

### 3.5 Study outcomes and endpoints

The primary endpoint of the study was to compare the incidence of dyskinesia and ICDs in patients with PD prescribed R‐PR to those prescribed IR‐DA. Dyskinesia and ICDs were identified using Read codes that were specified a priori and underwent review by a clinical coder and a neurologist. ICDs were classified as compulsive eating, pathological gambling, hypersexuality and other ICDs. The date of diagnosis of dyskinesia and ICDs was determined by the date of the earliest Read code.

Secondary endpoints included treatment adherence and persistence and time to levodopa initiation. Adherence to medication was measured by the medication possession ratio (MPR), defined as the ratio of the sum of the days' supply of the medicine between the first and last prescription date and the number of days in the same time period. In addition to the mean, MPR was categorised as follows: <30%, 30%‐50%, 51%‐80% and ≥80%. Treatment persistence was defined as the number of days between the initiation (date of first prescription) and discontinuation (date of last dose of medication).

### 3.6 Covariates

The following potential risk factors were considered in the analysis: calendar year of treatment initiation, PD duration and prior use of DAs at treatment initiation. These variables were either not included in the generation of the propensity score or did not achieve good balance following matching. In addition, age at PD diagnosis and gender were forced into the models since these were deemed to be clinically important variables.

### 3.7 Sample size determination

Based on a Cox regression of the log hazard ratio on a covariate with a SD of 0.50, assuming independent sampling of the exposure cohorts and an event rate (of dyskinesia) of 0.2, a sample‐size of 1000 patients (500 in each cohort) was required to achieve 82% power at a 0.05 significance level to detect a regression coefficient equal to 0.4055. We were not able to take account of the matching aspect of the design in our sample size calculation. It was anticipated that matching would reduce bias and potentially give the study some additional power.

### 3.8 Statistical analyses

Comparison of continuous variables between the R‐PR and IR‐DA cohorts was evaluated using the Student's *t* test for normally distributed variables (data presented as mean ± SD) and the Wilcoxon rank test for non‐normally distributed (skewed) variables (data presented as median, interquartile range [IQR]). Comparison of categorical variables was made using a chi‐squared test with Yates's correction or by Fisher's exact test depending on the sample sizes.

For dyskinesia and ICDs, the incidence rates per 1000 patient‐years, with 95% CI, for the matched R‐PR and IR‐DA cohorts were estimated from a Poisson regression model using the SAS PROC GENMOD procedure. The incidence rate ratio (R‐PR vs IR‐DA, with 95% CI) was also estimated. We used a Cox regression to estimate the hazard ratio and 95% CI of time to levodopa initiation in the two cohorts (R‐PR and IR‐DA). We verified the proportional hazards assumption by plotting Schoenfeld residuals against functions of time and testing for significance of time interaction terms with each predictor in the model.

All the potential confounders were included as adjustment variables in the multivariate Cox regression, and the matched cohorts were defined as a stratum variable in the SAS PROC PHREG procedure.

The Wilcoxon two‐sample test was used to compare the treatment persistence between the treatment cohorts.

## 4 RESULTS

**Figure 1:**
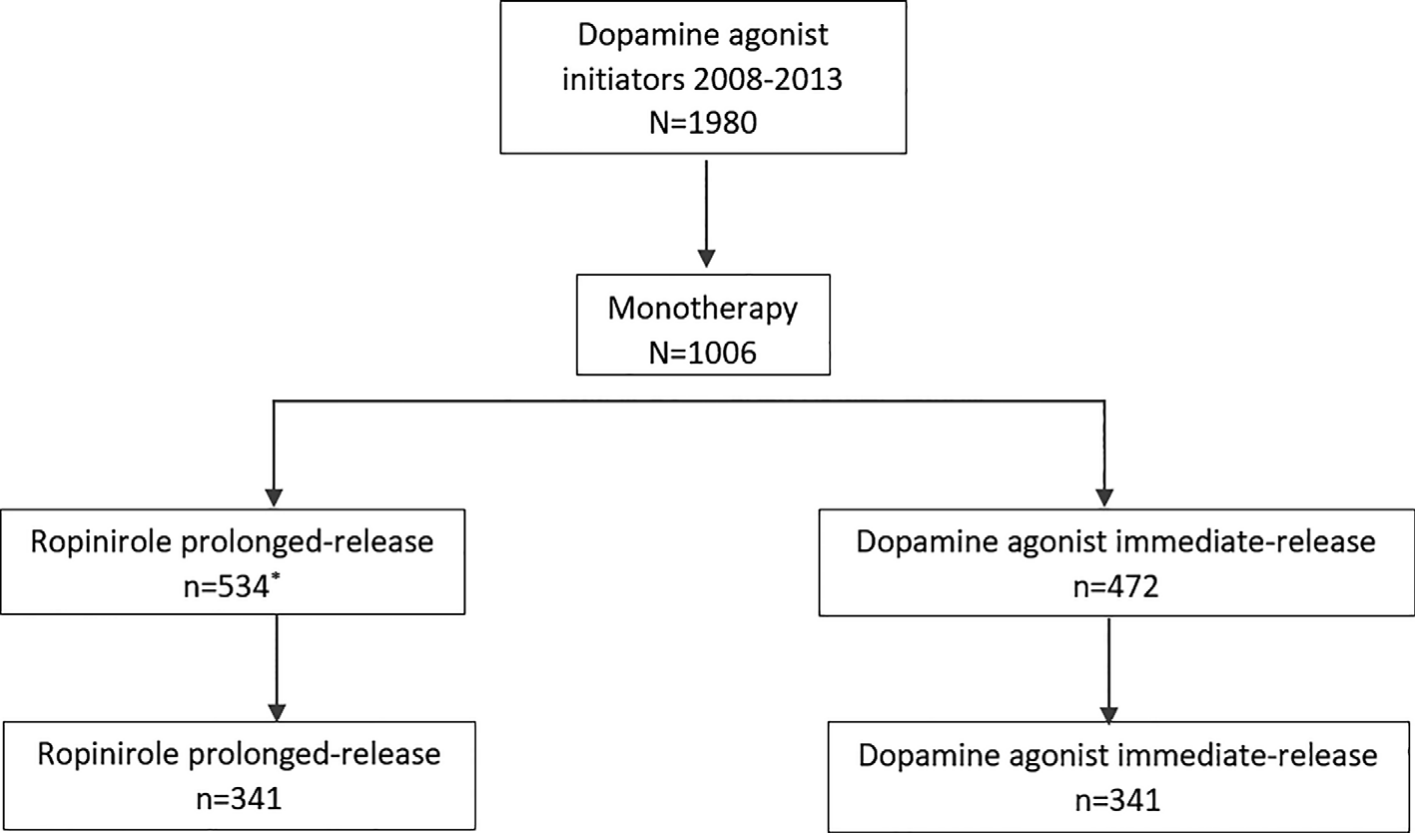
Study population. *Includes 324 switched to ropinirole prolonged‐release from the immediate‐release dopamine agonist initiator cohort CPRD, Clinical Practice Research Datalink

A total of 1980 patients with primary PD who initiated a DA between 2008 and 2013 were identified on the CPRD. Of these, 1006 patients (534 R‐PR and 472 IR‐DA) were prescribed a DA monotherapy. Following propensity score matching, 341 patients remained in each cohort (Figure 1 **)**.

### 4.1 Baseline characteristics after propensity score matching

Baseline characteristics of patients with PD before and after matching are shown in Table 1. Briefly, the R‐PR cohort after matching comprised 62.2% men, mean age at PD diagnosis was 64.1 years and mean duration from diagnosis to treatment initiation of PD was 8.7 months. The treatment cohorts were generally comparable after matching with regard to age at PD diagnosis, gender, average disease duration, Charlson comorbidity score, GP consultations in 12 months prior to treatment initiation and other anti‐Parkinson's drugs (MAO‐B inhibitors, amantadine, apomorphine, Catechol‐O‐methyl transferase [COMT] or decarboxylase inhibitors) prior to treatment initiation. However, differences remained for some covariates including the proportion of patients with a history of diabetes (R‐PR vs IR‐DA: 7.3% vs 3.8%; *P* = .05). Those variables that were heavily channelled towards R‐PR treatment (prior use of DA and calendar year of treatment initiation) were not included in propensity score calculation, but were adjusted for in the analysis.

**Table 1:** Baseline characteristics of ropinirole prolonged‐release and immediate‐release dopamine agonist cohorts (before and after propensity score matching)

	Before matching			After matching		
	R‐PR n = 534	IR‐DA n = 472	*P* value	R‐PR n = 341	IR‐DA n = 341	*P* value
Sex						
Men, n (%)	338 (63.3)	292 (61.9)	.64	212 (62.2)	211 (61.9)	.94
Women, n (%)	196 (36.7)	180 (38.1)		129 (37.8)	130 (38.1)	
Age at PD diagnosis (years), Mean (95% CI)^a^	62.8 (62.0‐63.5)	65.3 (64.5‐66.1)	<.0001	64.1 (63.2‐65.1)	63.5 (62.5‐64.5)	.34
Age at initiation of therapy, Mean (95% CI) ^a^	64.1 (63.4‐64.9)	65.8 (65.0‐66.7)	.003	64.9 (64.0‐65.8)	64.2 (63.2‐65.2)	.31
Duration from diagnosis to treatment initiation (months), Mean (95% CI), Median (IQR: P25, P75)^a^	15.9 (14.4‐17.5), 9 (3, 25)	6.2 (5.2‐7.3), 1 (0, 6)	<.0001^b^	8.7 (7.4‐10.0), 4 (1, 10)	8.1 (6.8‐9.5), 2 (1, 11)	.57
Charlson comorbidity score, Mean (95% CI)^a^	0.7 (0.6‐0.8)	0.8 (0.7‐0.9)	.27	0.7 (0.6‐0.9)	0.7 (0.6‐0.8)	.55
GP consultations in 12 months prior to initiation, Mean (95% CI)^a^	10.3 (9.5‐11.0)	10.9 (10.2‐11.7)	.20	10.9 (9.9‐11.9)	11.1 (10.3‐12)	.73
Other DAs prior to initiation, Mean (95% CI)^a^	0.7 (0.7‐0.8)	0.1 (0‐0.1)	<.0001	0.6 (0.6‐0.7)	0.1 (0‐0.1)	<.0001
Other anti‐Parkinson's drugs prior to initiation^c^ Mean, (95% CI)^a^	0.2 (0.2‐0.3)	0.2 (0.1‐0.2)	.006	0.2 (0.1‐0.2)	0.2 (0.2‐0.2)	.71
Propensity score, Mean (95% CI)^a^	0.6 (0.6‐0.6)	0.5 (0.5‐0.5)	<.0001	0.5 (0.5‐0.5)	0.5 (0.5‐0.5)	.94
History of diabetes, N (%)^d^	35 (6.6)	51 (10.8)	.02	25 (7.3)	13 (3.8)	.05
First DA used following PD diagnosis, N (%) patients^d^	163 (30.5)	446 (94.5)	<.0001	129 (37.8)	319 (93.5)	<.0001

Abbreviations: CI, confidence interval; COMT, Catechol‐O‐methyl transferase; DA, dopamine agonist; IQR, interquartile range; IR‐DA, immediate‐release dopamine agonist; GP, general practitioner; MAO‐B, monoamine oxidase; PD, Parkinson's disease; R‐PR, ropinirole prolonged‐release.

a Student's *t* test was performed for normally distributed variables (data presented as mean ± SD).

b Wilcoxon rank test for non‐normally distributed (skewed) variables (data presented as median, IQR).

c Amantadine, apomorphine, COMT inhibitor or Decarboxylase inhibitor, MAO‐B inhibitor.

d Comparison of categorical variables was made using a chi‐squared test with Yates's correction or by Fisher's exact test depending on the sample sizes.

### 4.2 Assessment of primary endpoints

Less than five events of dyskinesia were identified during the follow‐up period. The incidence of dyskinesia did not significantly differ between the R‐PR and the IR‐DA cohorts (incidence rate ratio: R‐PR vs IR‐DA: 0.76 [95% CI: 0.11‐5.38]) (Table 2). All patients with dyskinesia had initiated levodopa therapy during the observation period, prior to the dyskinesia diagnosis. Fewer than five cases of ICD were identified, all of which occurred in the IR‐DA cohort **(**Table 2 **)**.

**Table 2:** Incidence of dyskinesia occurrence in ropinirole prolonged‐release and immediate‐release dopamine agonist cohorts (after propensity score matching)

	R‐PR n = 341	IR‐DA n = 341
Incidence of dyskinesia per 1000 PY (95% CI)	2.98 (0.74‐11.9)	3.93 (0.98‐15.7)
Incidence rate ratio (IR‐DA vs R‐PR) (95% CI)	0.76 (0.11‐5.38)	
ICD, n	0	<5 cases

Abbreviations: CI, confidence interval; ICD, impulse control disorder; IR‐DA, immediate‐release dopamine agonist; PY, person‐years; R‐PR, ropinirole prolonged‐release.

### 4.3 Assessment of secondary endpoints

The patients in the R‐PR cohort remained on treatment for a significantly longer duration than those in the IR‐DA cohort (682 days vs 444 days; *P* < .0001) (Table 3). The proportion of patients considered adherent (≥80% MPR) was significantly higher in the R‐PR than the IR‐DA cohort (68.6% vs 45.7%; *P* < .0001). The mean MPR was also significantly higher (76.9 vs 64.4; *P* < .0001) in the R‐PR vs IR‐DA cohort **(**Table 3 **)**.

**Table 3:** Analysis of secondary endpoints for ropinirole prolonged‐release and immediate‐release dopamine agonist cohorts (after propensity score matching)

	R‐PR n = 341	IR‐DA n = 341
Adherence and persistence		
Duration of treatment (days), median (IQR)	682 (309, 1099)*	444 (147, 848)*
Duration of treatment (days), mean (95% CI)	748.4 (693.9‐802.9)	605.9 (548.7‐663.0)
MPR, n (%):		
<30%	56 (16.4)	68 (19.9)
30%‐50%	10 (2.9)	39 (11.4)
51%‐80%	41 (12.0)	78 (22.9)
≥80%	234 (68.6)	156 (45.7)
MPR, mean (95% CI)	76.9 (73.2‐80.6)*	64.4 (60.6‐68.1)*
Time to levodopa initiation		
Number of patients who initiated levodopa, n (%)	159 (46.6)	138 (40.7)
Time to initiation of levodopa (days), median (IQR)	417 (205, 736)	297 (111, 552)
Time to initiation of levodopa (days), mean (95% CI)	500.6 (444.3‐556.8)	399.6 (336.0‐463.1)

Abbreviations: CI, confidence interval; IQR, interquartile range; IR‐DA, immediate‐release dopamine agonist; MPR, medication possession ratio; R‐PR, ropinirole prolonged‐release.

* *P* < .0001.

The crude median time to levodopa initiation (ie, calculated directly from the data, not estimated by the survival model) was 417 days (IQR: 205‐736) in R‐PR vs 297 days (IQR: 111‐552) in IR‐DA cohort; however, the difference was not significant **(**Table 3 **)**. The adjusted hazard ratio for R‐PR vs IR‐DA for initiating levodopa, assessed by the Cox PH model, was 0.7 (95% CI: 0.37‐1.31; *P* = .26).

## 5 DISCUSSION

We evaluated the long‐term outcomes among patients with PD prescribed R‐PR compared to a propensity score matched cohort prescribed IR‐DA, using retrospective electronic healthcare data from UK primary care. Although the primary objective was to evaluate the incidence of dyskinesia, the number of events was low and the study therefore did not have sufficient power for this outcome. The incidence of dyskinesia in the R‐PR group was 2.98 per 1000 person‐years compared to 3.93 per 1000 person‐years in the IR‐DA cohort; however, this difference was not significant. The patients in the R‐PR cohort remained on treatment for a significantly longer duration and had greater adherence to medication than those in the IR‐DA cohort. Although a delay in time to levodopa initiation was observed in R‐PR vs IR‐DA cohort, this difference was not statistically significant.

In this study, individuals with PD were identified using Read codes. A pilot study conducted by GlaxoSmithKline (GSK) (unpublished data) found that 97.5% of the cases identified by Read code were confirmed to be PD based on responses from GP questionnaires. Furthermore, findings from the pilot study showed that by using the earliest of a prescription for a dopaminergic therapy, or Read code for PD or PD symptom, the assignment of PD diagnosis date was ascertained more accurately than by using the date of first PD Read code alone. The pilot study also evaluated the agreement between identifying dyskinesia and ICD using the Read codes vs data provided from the GP questionnaire; approximately 60% of dyskinesia and all cases of ICD in the pilot study were correctly identified.

In the current study, the cumulative incidence of dyskinesia was less than 1% for both R‐PR and IR‐DA cohorts. This is lower than that reported by previously published studies of ropinirole, which report estimates ranging from 3% to 52%.^8^, ^17^, ^18^, ^19^, ^20^ The risk of dyskinesia increases with PD severity, duration and dose of levodopa treatment.^21^ Other risk factors include younger age of PD onset, female gender and weight.^22^, ^23^

Most of the patients in this study had a recent PD diagnosis and were therefore earlier in their PD disease course. We included only those patients who were initially prescribed DA as monotherapy, and individuals with prior use of levodopa were excluded. The median time to dyskinesia onset in levodopa‐treated patients with PD has been reported to range from 4 to 6.5 years.^24^ In a US cohort study, the median time to dyskinesia onset from levodopa initiation was 4 years.^25^ In our study, the median period of follow‐up from the index date was 1.9 and 1.2 years for R‐PR and IR‐DA, respectively, and the median time to initiation of levodopa in our treatment cohorts was 1.1 and 0.8 years, respectively. Although all the patients who had developed dyskinesia had also initiated levodopa prior to the onset of dyskinesia, the relatively short period of follow‐up and limited exposure time to levodopa may in part explain the lower observed rates of dyskinesia in this study.

It is also important to acknowledge the possibility of under‐reporting or under‐diagnosis of dyskinesia in the primary care setting. For example, there may be a lack of recognition of dyskinesia in primary care. Another possibility is that a dyskinesia diagnosis made by a specialist was not reported back or recorded in structured data on the primary care record. Instead, it may have existed as free text or in discharge summaries.

Patients with PD receiving a DA medication are reported to be at increased risk of developing ICDs including pathological gambling and hypersexuality.^26^ As reported by previous studies, the prevalence of ICDs in patients with PD ranges from 6% to 39%.^27^, ^28^, ^29^ In the present study, a very small number of ICD cases were identified and all occurred in the IR‐DA cohort. The assessment of ICDs in published studies is heterogeneous and self or caregiver surveys or interviews are more likely to yield higher estimates of ICD prevalence than data recorded as part of routine clinical care. Furthermore, patients or family members may be reluctant to voluntarily report or consult their primary care physician for ICDs since some behavioural changes may be of a particularly sensitive nature. Factors such as longer disease duration and presence of motor complications are risk factors for developing ICDs.^30^ The lower risk of ICDs observed could be due to the relatively newly diagnosed PD population evaluated in our study and to the limited follow‐up.

Treatment persistence and MPR were both significantly higher for the R‐PR cohort than the IR‐DA cohort. It is possible that the higher MPR is due to the simpler dosing regimen (once a day) of R‐PR compared to IR‐DA.^31^, ^32^

Lastly, median time to levodopa initiation was slightly greater (though not significant) in the R‐PR cohort compared to the IR‐DA cohort; however, the study was not powered to detect such an effect.

A major strength of this study was the use of a real‐world group of PD patients with prescriptions and diagnoses that were recorded as part of routine clinical primary care. We used the CPRD, which is considered to be broadly representative of the UK population and has been widely used in pharmacoepidemiology studies.

The use of administrative data of this type carries some limitations including our limited ability to control for confounding. However, we used propensity score matching to improve comparability between the exposure cohorts at baseline. A number of factors such as PD severity and nature of the PD symptoms, some of which are not available on the CPRD, may influence GP's choice of therapy. Instead, we included information such as disease duration, healthcare utilisation prior to treatment initiation and Charlson score as proxy measures in the estimation of propensity score. Whilst this did improve comparability, differences remained for some covariates. Individuals in the R‐PR cohort were more likely to have switched from another DA, whereas the IR‐DA initiators were more likely to have initiated therapy as a first‐line DA treatment. The reasons for switching are not known; however, it could reflect a subset of patients, who were not optimally controlled for their PD symptoms, did not tolerate their previous medication, or experienced an adverse event. The impact of this difference on the observed outcomes under study is not known.

In addition, it is possible that the initial prescription at the time of PD diagnosis was made by a PD specialist and not a GP and therefore, may not have been recorded in structured fields on the CPRD. As previously mentioned, GPs may not be as familiar with ICDs or dyskinesia as PD specialists, and this could result in miscoding or a failure to recognise these events. Further, GPs may change the dose or medication to deal with adverse consequences rather than record it as a diagnosis.

We used prescriptions issued by the GP as a proxy for treatment adherence. This merely represents prescriptions given to the patient and does not represent dispensed prescriptions. It is therefore possible that some patients were not taking their medications but were still receiving prescriptions. Although this may overestimate adherence, it is not expected to account for the differences observed between the groups. Finally, the study did not account for the doses of dopaminergic medication prescribed to the patients or medications given along with levodopa to prolong its action (eg, COMT, decarboxylase or MAO‐B inhibitors).

## 6 CONCLUSIONS

Although the power achieved in this study was low due to lower than expected number of dyskinesia and ICD events, some clinically relevant trends were observed.

Patients who received R‐PR remained on treatment significantly longer and had greater medication adherence than patients receiving IR‐DA. A non‐significant delay in levodopa initiation was observed in the R‐PR cohort in comparison to the IR‐DA cohort; however, this must be interpreted with caution since the study was not adequately powered to evaluate this.

## ETHICS STATEMENT

Trial registration number: 111 981 (GlaxoSmithKline Trial registry; https://www.gsk-studyregister.com/).

## CONFLICT OF INTEREST

U.G., O.K. and N.G. are current salaried employees of GSK with stock holdings. C.E.B. was a GSK employee with stock holdings during study duration.

## AUTHOR CONTRIBUTIONS

U.G. contributed to the conception and design of the study, data acquisition, data analysis and interpretation. O.K. and C.E.B. contributed to the data acquisition and data analysis and interpretation. N.G. contributed to the conception and design of the study and data analysis and interpretation. All authors made critical revisions to the draft versions of manuscript and approved the final manuscript.

## Data Availability

Anonymised individual participant data and study documents can be requested for further research from www.clinicalstudydatarequest.com.
